# The Blood-Brain Barrier and the EphR/Ephrin System: Perspectives on a Link Between Neurovascular and Neuropsychiatric Disorders

**DOI:** 10.3389/fnmol.2018.00127

**Published:** 2018-04-12

**Authors:** Victoria A. Malik, Barbara Di Benedetto

**Affiliations:** ^1^RG Neuro-Glia Pharmacology, Department of Psychiatry and Psychotherapy, University of Regensburg, Regensburg, Germany; ^2^Regensburg Center of Neuroscience, University of Regensburg, Regensburg, Germany

**Keywords:** blood-brain barrier, EphR/ephrin, endothelial cells, astrocytes, neurovascular disorders, neuropsychiatric disorders

## Abstract

Interactions among endothelial cells (EC) forming blood vessels and their surrounding cell types are essential to establish the blood-brain barrier (BBB), an integral part of the neurovascular unit (NVU). Research on the NVU has recently seen a renaissance to especially understand the neurobiology of vascular and brain pathologies and their frequently occurring comorbidities. Diverse signaling molecules activated in the near proximity of blood vessels trigger paracellular pathways which regulate the formation and stabilization of tight junctions (TJ) between EC and thereby influence BBB permeability. Among regulatory molecules, the erythropoietin-producing-hepatocellular carcinoma receptors (EphR) and their Eph receptor-interacting signals (ephrins) play a pivotal role in EC differentiation, angiogenesis and BBB integrity. Multiple EphR-ligand interactions between EC and other cell types influence different aspects of angiogenesis and BBB formation. Such interactions additionally control BBB sealing properties and thus the penetration of substances into the brain parenchyma. Thus, they play critical roles in the healthy brain and during the pathogenesis of brain disorders. In this mini-review article, we aim at integrating the constantly growing literature about the functional roles of the EphR/ephrin system for the development of the vascular system and the BBB and in the pathogenesis of neurovascular and neuropsychiatric disorders. We suggest the hypothesis that a disrupted EphR/ephrin signaling at the BBB might represent an underappreciated molecular hub of disease comorbidity. Finally, we propose the possibility that the EphR/ephrin system bears the potential of becoming a novel target for the development of alternative therapeutic treatments, focusing on such comorbidities.

## Introduction

Structural integrity of the blood-brain barrier (BBB) is essential for the establishment and maintenance of brain homeostasis. Any disruption in its cellular or structural components may exert devastating effects on mental health. During the past couple of years, research on the neurovascular unit (NVU) has experienced a second revival. This highlighted the BBB as a potential novel target for the development of alternative treatment strategies for brain and vascular pathologies.

### Structural Components and Function of the BBB

**Figure 1:**
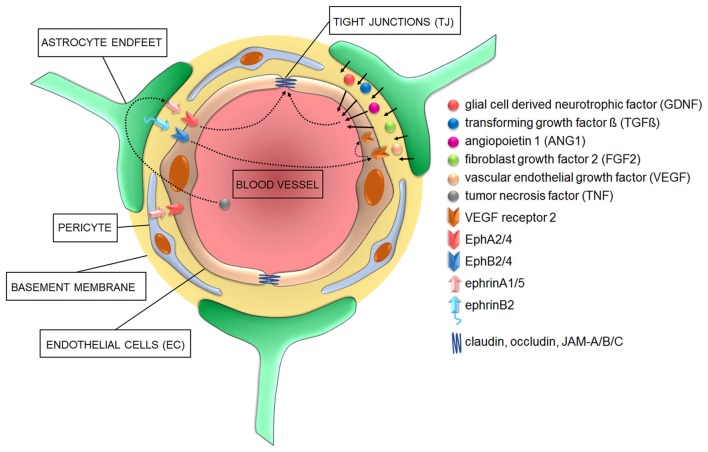
Cellular and signaling components of the blood-brain barrier (BBB) in health conditions. Astrocyte-derived signaling molecules influence the development and/or maintenance of BBB properties. Additionally, various members of both “A” and “B” classes of the ephrin family of ligands located on either astrocyte and/or pericytes do activate EphA and EphB receptors on endothelial cells (EC) to influence EC differentiation during angiogenesis and the development of tight junctions (TJ) during barriergenesis.

The BBB is a multicellular vascular structure separating the central nervous system from peripheral blood circulation (Obermeier et al., 2013). It is composed of cerebrovascular endothelial cells (EC) forming brain vessels, astrocytes and extracellular matrix (ECM) components providing structural support (Abbott et al., 2006). Pericytes are also relevant to form the BBB and their functional roles have been fully described elsewhere (Cabezas et al., 2014; Figure 1). Together, all these elements exert their functions as a selective physical (Abbott et al., 2006), transport (Begley and Brightman, 2003) and metabolic (Pardridge, 2003, 2016) barrier, tightly controlling the passage of molecules in and out of brain parenchyma and preventing the penetration of toxins or pathogens (Obermeier et al., 2013).

### Endothelial Cells

Cerebral EC have a unique characteristic in comparison to peripheral EC: they are interconnected by continuous intracellular multiprotein complexes called tight junctions (TJs), which lack fenestrations and undergo extremely low rates of transcytosis (Figure 1). This limits paracellular passage of substances and directs molecular trafficking to take a rigorously controlled transcellular route across the BBB (Abbott et al., 2006). Such a strong physical barrier allows only small gaseous and lipophilic molecules to diffuse freely in and out of the brain, whereas bigger molecules need to be actively transferred via transporter/carrier systems, such as the glucose transporter-1 (GLUT-1) or the large neutral amino acid transporter-1 (LAT-1) located on the luminal (blood facing) or abluminal (brain facing) EC sites (Borst and Schinkel, 2013). Potentially harmful compounds like glutamate are actively cleared from the brain even against a concentration gradient requiring ATP as energy source (e.g., via excitatory amino acid transporter 1/2; EAAT1/2 (Hawkins and Viña, 2016). Generally, large hydrophilic molecules cannot be transferred across the BBB unless by specific receptor- or adsorptive-mediated transcytosis (Pardridge, 2003, 2016; Strazielle and Ghersi-Egea, 2016).

The TJ are key regulators of paracellular permeability and transendothelial electrical resistance. Major constituents of the TJ are transmembrane molecules like occludin (Yu et al., 2005), which links to the cytoskeleton via the accessory proteins zonula occludens (ZO-1/2) and claudins (Piehl et al., 2010), and junctional adhesion molecules (JAM-A, -B, -C, Mandel et al., 2012). During early embryogenesis, pre-existing vessels sprout and undergo angiogenesis (Obermeier et al., 2013). Sealing properties, including refinement of the protein complexes, establishment of efflux transporters and limitation of transcytosis, seems to only mature when sprouting vessels come in close contact with pericytes and astrocytes (Daneman et al., 2010; Obermeier et al., 2013). However, the role of astrocytes in this process is still a matter of controversy (see below). Afterwards, matured TJ are fixed and need to be maintained throughout life.

### Astrocytes

Astrocytes regulate features of the BBB through the tips of their processes, called astrocytic endfeet, which surround and contact brain micro-vessels (Kettenmann and Verkhratsky, 2008). Among other functions, they regulate the ion balance around the BBB and secrete and recycle neurotrophic factors necessary to control TJ (Gee and Keller, 2005). A very elegant example of how astrocytes maintain the ionic homeostasis is represented by their synchronized spatial K^+^ buffering at synaptic and BBB locations mediated by their perivascular and perisynaptic endfeet (Olsen and Sontheimer, 2008). This controls ion concentrations during normal brain activity and can thereby link and adapt responses of blood vessels to synaptic neuronal activity to guarantee the appropriate supply of oxygen and nutrients (Wolburg et al., 2011). Additionally, astrocytes secret several molecules such as the glia cell-derived neurotrophic factor (GDNF; Igarashi et al., 1999), transforming growth factor β (TGF-β; Dobolyi et al., 2012), angiopoietin 1 (ANG1; Easton, 2012), fibroblast growth factor 2 (FGF2; Reuss et al., 2003) and vascular endothelial growth factor (VEGF; Rosenstein et al., 2010) which act on EC to either promote TJ formation and/or regulate BBB permeability (Figure 1).

At present, it is still controversial whether astrocytes are necessary for the induction of TJ, because of the temporal shift between EC differentiation/maturation and astrocyte development. Recent work suggests that astrocytes are dispensable for the induction of TJ (Saunders et al., 2016), but are necessary for their further strengthening and maintenance throughout life (Alvarez et al., 2011, 2013). However, meningeal blood vessels which lack contacts with astrocytes display higher vascular permeability than EC-BBB, supporting indeed the necessity of astrocytes to induce BBB properties (Lécuyer et al., 2016).

### Basement Membrane

The non-cellular component of the NVU is the basement membrane, which is composed of structural proteins such as collagen-IV, laminin and fibronectin, among others (Cardoso et al., 2010; Figure 1). The main function of the basement membrane is to provide stability to the other members of the NVU and regulate their crosstalk enabled by matrix transmembrane receptors like integrins and dystroglycans (Baeten and Akassoglou, 2011).

## The Erythropoietin-Producing-Hepatocellular Carcinoma Receptors (EphR) and Eph Receptor-Interacting Signals (Ephrins) System

The erythropoietin-producing-hepatocellular carcinoma receptors (EphR)/Eph receptor-interacting signals (ephrin) system was first discovered in 1990, when ephrinA1 was characterized as a tumor necrosis factor (TNF)-inducible protein in human umbilical vein EC (HUVEC; Holzman et al., 1990). EphRs/ephrins typically mediate contact-dependent communication between cells to control cell fates. During development, this system plays an important role in spatial organization, axon guidance, formation of synaptic connections and blood vessel remodeling. In the adulthood, it mostly regulates synaptic remodeling, epithelial differentiation, bone remodeling, immune function, insulin secretion and stem cell self-renewal (Kullander and Klein, 2002; Yamaguchi and Pasquale, 2004; Pasquale, 2005, 2008).

Eph receptors comprise the largest family of receptor tyrosine kinases (RTK). Eph receptors and ephrins can be divided into subclasses A and B. In humans, nine EphA and five EphB receptors are known. They consist of an extracellular part including a globular ligand-binding domain, a cysteine rich region two fibronectin type 3 repeats and a cytoplasmic domain comprised of a short juxtamembrane region with several conserved tyrosine residues, a sterile alpha motive protein-protein interaction domain and a C-terminal PDZ binding motif. Additionally, several alternatively spliced forms have been identified with distinct functions (Pasquale, 2010). Ephrins, on the other hand, can be further distinguished by their membrane attachment: ephrinAs are anchored via a glycosylphosphatidylinositol (GPI) linkage, whereas ephrinBs are attached via a single transmembrane domain containing a short cytoplasmic PDZ-binding motif. EphRs and ephrins can be expressed on the same cell, in mutually exclusive expression patterns or in complementary gradients, establishing a highly dynamic signaling system (Lisabeth et al., 2013).

### The EphR/Ephrin System: Signaling Mechanisms

Besides the well-known bidirectional signaling activated upon cell-cell interactions, which is described in detail in some excellent reviews (Pasquale, 2008; Murai and Pasquale, 2011; Klein, 2012; Lisabeth et al., 2013), several alternative signaling mechanisms have been proposed for the EphR/ephrin system.

Upon receptor/ligand interaction, several downstream signaling cascades are activated to mediate cell adhesion or repulsion, depending on the type and abundance of ligands and receptors present on cell surfaces (Janes et al., 2012). These signaling pathways include, among others, the Src kinase family, mitogen-activated protein kinase, and integrin mediated pathways (Lackmann and Boyd, 2008; Pasquale, 2008; Pitulescu and Adams, 2010). Their activity is dependent on Rho family GTPases, including RhoA, Rac1, Cdc42 and a variety of guanine nucleotide exchange factors (GEF), like ephexins (Cowan et al., 2005; Pasquale, 2008). After the initial receptor/ligand interaction, intact EphR/ephrin complexes together with potentially associated cytoplasmic proteins and the surrounding membrane are internalized in either cell. This Rac1-dependent mechanism is termed trans-endocytosis and provides a mechanism to switch between cell adhesion and retraction fates and to terminate receptor signaling activity (Lisabeth et al., 2013).

Besides trans-endocytosis, the activation of enzymes which initiate proteolytic cleavage represents another alternative signaling mechanism (Atapattu et al., 2014). Among such enzymes, A disintegrin and metalloproteases (ADAM) and matrix metalloproteases (MMP) are implicated in signal termination (Atapattu et al., 2014).

In mammalian tissues, members of the ADAMs family are transmembrane metalloproteases able to process and shed ectodomains of membrane bound receptors (Klein and Bischoff, 2011). They play crucial roles in pathological conditions such as inflammation or stress-mediated angiogenic responses (Weber and Saftig, 2012). Several EphRs/ephrins of both A and B subclasses can associate with ADAMs resulting in their own cleavage. Cleavage of the ligand-bound receptor leads to a breakdown of the molecular tethers between interacting cells, thereby favoring the internalization of receptor/ligand complexes, as exemplified by ADAM10 initiated cleavage of the EphA3/ephrinA2 complex during axon detachment (Hattori et al., 2000; Mancia and Shapiro, 2005) or of the EphA2/ephrinA1 complex (Salaita et al., 2010).

MMPs cleave proteins located either on membranes or in extracellular spaces (Miller et al., 2008). Their main function is to degrade structural components of the ECM to facilitate cell migration (Streuli, 1999), especially during angiogenesis and inflammatory processes (Kessenbrock et al., 2010; Palmisano and Itoh, 2010). Recently, it has been shown that MMPs cleave ephrinA1 and ephrinA2 from their GPI-anchor, leading to the release of functional soluble monomers which can act on distant Eph receptors (Beauchamp and Debinski, 2012). Followed by an initial shedding step mediated by ADAMs or MMPs, EphRs/ephrins can further be processed by intramembrane cleaving proteases such as γ-secretase (Bergmans and De Strooper, 2010) or neuropsin (Attwood et al., 2011; Morohashi and Tomita, 2013). This events generates cytoplasmic active fragments (Litterst et al., 2007; Xu and Henkemeyer, 2009) which may i.e., regulate behavioral responses such as anxiety (Attwood et al., 2011).

The signaling cluster propagation represents another noteworthy alternative signaling mechanism to be mentioned. This type of signaling, originally initiated by receptor/ligand interactions in *trans*, causes the formation of lateral clusters through receptor-receptor interactions in *cis*. These receptor clusters do no longer rely on ephrin interaction to get activated, enabling the strong amplification of an originally small signal generated by a first short cell-cell contact (e.g., EphA3/ephrinA5; Wimmer-Kleikamp et al., 2004).

Such signaling effectors of the Eph/ephrin system might become relevant in brain disorders to identify alternative targets for drug discovery.

### The Role of the EphR/Ephrin System for the Development and Function of the Vasculature and the BBB

The interaction of specific cell types to properly develop the vascular system and a functional BBB is an essential process which requires the appropriate temporally- and spatially-regulated expression of distinct guidance cues. Among them, the EphR/ephrin system represents an ideal candidate to exert those functions.

During vasculogenesis, VEGF induces ephrinA1 expression which activates EphA2 on neighboring EC, thus exerting angiogenic effects—*in vitro* and *in vivo* (Cheng et al., 2002; Brantley-Sieders et al., 2004). Despite the previously mentioned controversy, astrocytes release VEGF during embryonic development and might therefore contribute to the early TJ formation. Later on in development, however, for the further differentiation of EC and formation of an efficient BBB, the inhibition of EphA2 activity in human brain micro-vessel EC (HBMEC) is instrumental to promote TJ strengthening (Zhou et al., 2011; Figure 1). These different functions mediated by the tightly controlled expression levels of EphA2 suggest that the regulation of EphA2 dosages may underlie the “switch” between early/angiogenic and late/barriergenic effects of EphA2 in EC. Moreover, they suggest that putative interactions between EphA2-expressing EC with ephrinA1-expressing perivascular astrocytes or pericytes may also control TJ formation in physiological conditions or their disruption during pathogenic processes (Lécuyer et al., 2016). In a different system, the pulmonary system, stimulation of arterial EC with ephrinA1 also increases their permeability (Larson et al., 2008), further supporting that the overexpression of certain EphR/ephrin interactions might influence barrier integrity, ultimately impacting brain homeostasis. Astrocytes express several other members of the EphR/ephrin system (Nestor et al., 2007) which may be relevant during both vasculogenesis and/or barriergenesis. For example, the proper interaction between EphA4/ephrinA5 located on EC and astrocyte endfeet, respectively, is necessary for the development of a normal vascular system in the hippocampus of adult mice (Hara et al., 2010). Additionally, radial glia cells provide a physical scaffold and chemical signals to support the very early stages of angiogenesis (Cheslow and Alvarez, 2016). Among such signals, EphA4 expressed on EC has been indicated to guide the invasion of the developing brain by newly forming micro-vessels in response to glial-dependent stimulation (Goldshmit et al., 2006).

With regard to the “B” types, ephrinB2 controls VEGF receptor (VEGFR)-2 internalization, which is necessary for receptor activation and VEGF-induced filopodial extension in EC during angiogenesis (Bochenek et al., 2010; Sawamiphak et al., 2010; Pitulescu and Adams, 2014). During these events, the role of the EphB2/ephrinB2 interaction is essential for blood vessel assembly (Foo et al., 2006). During cardiovascular development, EphB4/ephrinB2 signaling in EC is additionally activated to properly specify arterial vs. venous identity (Wang et al., 1998; Adams et al., 1999; Gerety et al., 1999; Gale et al., 2001; Augustin and Reiss, 2003).

## Disrupted Integrity of the BBB and the Eph/Ephrin System—The “Missing Link” Between Neurovascular and Neuropsychiatric Pathologies?

BBB leakiness is a hallmark of neurovascular pathologies comorbid with neuroinflammatory processes (Lee et al., 2009; Abbott and Friedman, 2012). Recent work has also evidenced signs of BBB leakiness in neuropsychiatric disorders, which are also accompanied by elevated levels of blood-circulating pro-inflammatory cytokines and TNF (Miller et al., 2009; Janelidze et al., 2011; Liu et al., 2012; Salim et al., 2012; Najjar et al., 2013). Furthermore, preclinical and clinical studies have evidenced a wide range of comorbidity between neurovascular and neuropsychiatric disorders with concurrent neuroinflammation (Dantzer et al., 2008; Wood, 2014; Hodes et al., 2015; Patel and Frey, 2015; Seligman and Nemeroff, 2015; Miller and Raison, 2016; Barnes et al., 2017; Menard et al., 2017), thereby suggesting that common neurobiological substrates may underlie such high degrees of comorbidities. In view of the regulatory roles of the EphR/ephrin system during the development and maturation of a proper BBB sealing properties, it appears evident how this system might be considered a hub of brain disorders associated with BBB disruption.

**Figure 2:**
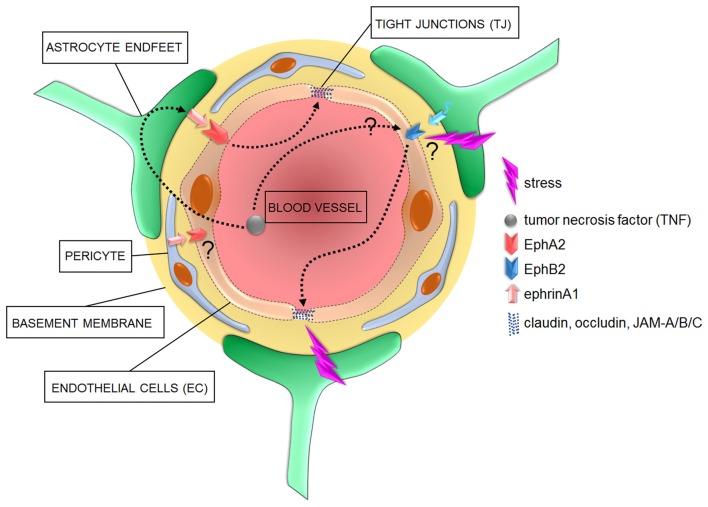
Cellular and signaling components of the BBB in pathological conditions. In diseased brains, the overactivation of astrocyte- or pericyte-dependent ephrin signaling may affect TJ via an increased activity of Eph receptors, with consequent increased barrier permeability to blood circulating inflammatory factors such as tumor necrosis factor (TNF). Increased stress has also been shown to correlate with a compromised barrier functionality and impaired TJ which might further induce the onset of neurological and neuropsychiatric disorders. However, specific molecular mediators of such effects are yet to be identified.

Preclinical studies indicated that, among “A” members of the EphR/ephrin system, especially the EphA2 receptor mediates inflammation during injury, ischemia and other chronic inflammatory conditions in various murine models of neurovascular disorders (Jellinghaus et al., 2013; Thundyil et al., 2013; Ende et al., 2014). Specifically, EphA2 receptor activation occurs after brain injury and contributes to inflammation by promoting BBB permeability (Thundyil et al., 2013). Interestingly, the promoter of ephrinA1, the highest affinity ligand for EphA2, is a target of the pro-inflammatory marker TNF (Ende et al., 2014). Furthermore, whereas TNF has angiogenic properties during early embryogenesis (Cheng and Chen, 2001; Munthe and Aasheim, 2002), it triggers BBB hyperpermeability in adult epithelial tissues via the activation of both EphA2 and EphA4 in EC, with induction or exacerbation of neurovascular disorders (Jellinghaus et al., 2013; Thundyil et al., 2013; Ende et al., 2014; Figures 1, 2). These evidences suggest that investigating the EphA2/ephrinA1 bidirectional signaling between interacting cellular partners at the BBB may reveal novel molecular triggers of comorbidity between inflammatory/neurovascular/neuropsychiatric disorders and indicate alternative targets of therapeutic interventions. Among other candidates, although in a specific subset of neuropsychiatric disorders, it was shown that the binding between ephrinA5 expressed on astrocytes and its corresponding EphA4 receptor on EC is increased in the hippocampus of a mouse model of temporal lobe epilepsy, which shows an increased development of micro-vessels with detrimental consequences on brain homeostasis (Shu et al., 2016). Interestingly, the selective blockade of the EphA4/epHRin interaction is sufficient to attenuate the disease phenotype, further supporting the therapeutic relevance of a selective targeting of the EphR/ephrin system for neurovascular/psychiatric disorders.

Regarding the “B” members, a link between TNF and EphB2 has also been suggested to be relevant to induce inflammatory pathways (Pozniak et al., 2014; Figure 2). It has also been shown that EphB2 activity regulates cognitive functions and resilience or vulnerability to stress (Yuferov et al., 2013; Zhang et al., 2016). Among triggers of neuropsychiatric disorders, stress is one of the most detrimental (Charney and Manji, 2004). In line with this, as little as 2 days of stress provokes morphological changes in EC, accompanied by dysregulation of claudin-5 and occludin expression (Figure 2). These changes are paralleled by a decreased expression of GFAP, indicating an additional negative impact on astrocytes (Sántha et al., 2016). A clinical and a preclinical study also confirmed an astrocyte impaired phenotype, with reduced coverage of blood vessels by AQP-4-enriched astrocyte endfeet, in the prefrontal cortex of depressive patients and of a validated animal model of depression (Rajkowska et al., 2013; Di Benedetto et al., 2016). Moreover, a new study has clearly demonstrated the detrimental effects of stress on the permeability of BBB, with a reduction in its sealing properties (Menard et al., 2017). It would be interesting to investigate whether EphB2 might represent a molecular link between the above-mentioned stress-dependent changes in specific cellular components of the BBB, in its sealing properties and the modulatory effects on behavioral phenotypes.

A proof-of-principle that the EphR/ephrin system might represent a highly relevant therapeutic target for comorbid neurovascular and neuropsychiatric disorders has been provided by the controlled reactivation of EphB4/ephrinB2 in cardiovascular disorders, which enhanced BBB repair mechanisms (Ghori et al., 2017). This approach sounds promising to rescue BBB deficits and may putatively be beneficial to reverse comorbid maladaptive behavioral phenotypes.

## Conclusion

Although several studies provide evidence for a compromised BBB integrity in a broad variety of psychopathologies, it is still unknown whether the BBB disruption is a cause or a consequence of the disease. Depending on the circumstances, a transient opening of the BBB might even be beneficial, e.g., during inflammation, it may allow the passage of growth factors or antibodies to hinder the inflammatory process. On the other hand, tightening of the BBB appears necessary during periods of stress or hypoxia (Abbott et al., 2006).

Investigating the expression of EphR/ephrin system in the single components of the BBB during vasculogenesis and barriergenesis as well as their interplays in health and in the pathogenesis of brain and neurovascular disorders might open new avenues to understand neurobiological underpinnings of pathological comorbidities. This may help to identify novel therapeutic targets especially beneficial for comorbid patients.

## Author Contributions

VAM and BDB contributed to literature screening and drafting the manuscript; both authors read and approved the final version of the manuscript.

## Conflict of Interest Statement

The authors declare that the research was conducted in the absence of any commercial or financial relationships that could be construed as a potential conflict of interest.
